# Hiccup Relief Using Active Prolonged Inspiration

**DOI:** 10.7759/cureus.53045

**Published:** 2024-01-27

**Authors:** Stephen K Stacey, Michael S Bassett

**Affiliations:** 1 La Crosse-Mayo Family Medicine Residency Program, Mayo Clinic Health System, La Crosse, USA

**Keywords:** inspiration, case-series, hypoxia and hypercapnia, deep-inspiration breath-hold, mechanism and pathway for hiccups, acute hiccups, persistent hiccups, intractable hiccups

## Abstract

Background

Hiccups are a common physiologic reflex resulting from intermittent and involuntary spasmodic contraction of the diaphragm and intercostal muscles. While most cases are self-limited, lasting less than 48 hours, rare pathologies may result in prolonged symptoms. Hiccups can be disruptive and uncomfortable, leading many to seek management strategies using common home remedies. Few methods for terminating hiccups have been published in the scientific literature. We report the efficacy of the Hiccup relief using Active Prolonged Inspiration (HAPI) technique, which combines phrenic and vagal nerve stimulation with transient hypercapnia for hiccup relief.

Methods

Twenty patients with self-limited hiccups and one patient with prolonged hiccups were successful in eliminating hiccups using HAPI. In this method, patients are instructed to inspire maximally. Once at the peak of inspiration, they continue to attempt to inspire with an open glottis for a total of 30 seconds. This is followed by a slow expiration and resumption of normal respiration.

Results

In all cases, patients reported immediate hiccup relief.

Conclusion

These findings suggest the HAPI technique is a simple and viable method for hiccup relief. Further studies are needed to validate effectiveness.

## Introduction

Hiccups are a nearly universal phenomenon experienced by humans, even as early as in utero [1,2]. They are caused by involuntary myoclonic contraction of the diaphragm combined with intercostal contraction and a sudden closure of the glottis [3]. They are also occasionally referred to by the medical term singultus from a Latin term meaning “sobbing” [4]. The classic “hic” sound is created when the sudden inspiration is interrupted by the closing glottis [5].

The precise trigger of hiccups is unknown. Once an episode of hiccups begins, they typically occur at a rate of 40-60 per minute [6]. Spells of fetal hiccuping last an average of eight minutes, though the duration of adult spells is less clear [1]. The ubiquitous and transient nature of hiccups makes it difficult to determine how frequently they occur in adults. In a retrospective review of over 100,000 hospitalized patients, only 54 charts documented hiccups troubling enough to warrant medical attention [7].

It is unclear whether hiccups serve a beneficial function. It is hypothesized that the hiccup reflex is an evolutionary remnant of primitive respiratory mechanisms, and may aid fetuses in strengthening respiratory muscles [3]. Other theories suggest it is a method to eliminate unwanted gastric contents to aid in neonatal feeding [6]. No prevailing theories suggest a benefit for hiccups in adults, and rarely hiccups may even represent underlying pathology such as lesions in the brain or near the diaphragm.

Hiccups are a complex, coordinated neurologic process. The underlying neurologic reflex arc involves afferent impulses from the phrenic and vagus nerves, as well as the sympathetic chain. Efferent impulses are sent from the phrenic nerve to the diaphragm, stimulating unilateral or bilateral contractions. Action of the accessory nerve stimulates intercostal muscles, and the recurrent laryngeal branch of the vagus nerve stimulates glottal closure [3,8]. Without this last step, hiccups would lead to hyperventilation, as occurs in hiccuping patients with a tracheostomy [4].

The overwhelming majority of episodes of hiccups are a self-limited minor annoyance, lasting less than 48 hours. Hiccups that last more than 48 hours are considered persistent, and when they last more than one month they are considered intractable [4,8].

Most often, the cause of hiccups is idiopathic. Etiologies that have been identified include central nervous system disorders, medications, and irritants in the head, neck, and thoracoabdominal regions. Central nervous system disorders that can lead to hiccups include benign causes such as emotional stress or excitement, to rare but severe causes such as infarction, mass effect, demyelinating disease, syringomyelia, aneurysm, and other structural diseases. Medications that may trigger hiccups include benzodiazepines, steroids, chemotherapy agents, and barbiturates. Irritants can cause hiccups by affecting the diaphragm or branches of the vagus or phrenic nerves. Such causes can include gastric distention, peptic ulcer disease, gastritis, mediastinal masses, neck masses, and intraabdominal diseases [4,8].

Multiple home remedies for the treatment of hiccups have gained popularity through informal sharing and are generally considered safe and well-tolerated [4,9]. Examples of these techniques include breath-holding, Valsalva maneuver, rebreathing into a paper bag, inducing fear, and drinking water upside-down. Despite their widespread use and anecdotal success, the efficacy of these maneuvers has not been rigorously established. Pharmacological interventions have been examined, and observational data suggests gabapentin and chlorpromazine may be effective. Use of baclofen and metoclopramide is supported by small randomized, placebo-controlled trials [4,9].

One mechanism underlying many of these resolution techniques may be inhibition of involuntary activation of the neurologic reflex arcs [8]. Another mechanism that has demonstrated effectiveness is hypercapnia. Intractable hiccups are found to cease when hypercapnia is stimulated in the lab. Episodes of breath-holding and transient fear may be able to create conditions of hypercapnia sufficient to stop hiccups [10,11].

If conservative and medical therapies fail, more invasive approaches may be attempted which focus on interruption of the neurologic reflex arc. Phrenic nerve blocks have been used with success in numerous case reports, though prospective randomized data are not available [12-14]. This technique is often performed percutaneously under ultrasound guidance, and side effects appear to be uncommon. Vagal nerve stimulation has likewise been used successfully to cure intractable hiccups [15]. There is no good evidence to determine precise cure rates with these therapies, and it is known that neither technique is universally successful [16].

This case series presents the results of using a method to alleviate the hiccups known as the Hiccup relief using Active Prolonged Inspiration (HAPI) technique. This technique is designed to address both hypercapnia and interruption of the neurologic reflex arc through noninvasive means. In the HAPI technique, patients interrupt the neurologic reflex arc through conscious continuous activation of respiratory muscles as they perform continuous inspiration. The consequent breath-holding during this period also induces hypercapnia. It is hypothesized that the concurrence of both phenomena simultaneously may induce greater success than either alone.

## Materials and methods

This study is a retrospective case series evaluating 21 patients, both male (n=12) and female (n=9) Caucasians aged 17-64. Cases were collected over a span of eight years from 2016-2023. They were selected as a convenience sample of individuals who were incidentally observed during visits with the authors to have spontaneous episodes of hiccups. They were treated immediately and the success or failure of the technique was noted. Demographic data including age and gender were recorded for all patients without any personally identifiable information in a document on a password-protected computer. Patients were generally healthy, though background data on past medical history was not systematically obtained.

Most cases were of self-limited hiccups, lasting less than 24 hours. One case was a 42-year-old male who reported persistent hiccups for four days. He was otherwise healthy with no significant past medical history. He exercised regularly, took no medications, and obtained all routine preventive medical care. He reported unsuccessfully trying to eliminate the hiccups by holding his breath.

Each patient was educated on the performance of the HAPI technique (Table 1). They were instructed to inspire maximally then, once at peak inspiration, to continue to attempt to inhale for a total of 30 seconds. After 30 seconds, patients slowly exhale, and then resume normal respiration. When performing this technique, the patient contracts the diaphragm maximally, providing constant stimulus to the phrenic nerve. This is in contrast to classic breath-holding which allows the diaphragm to relax against a closed glottis.

**Table 1:** Steps of the Hiccup relief using Active Prolonged Inspiration (HAPI) technique

Steps of the Hiccup relief using Active Prolonged Inspiration (HAPI) technique
1. Inspire maximally
2. Once at peak inspiration, continue to attempt inspiration with an open glottis for a total of 30 seconds
3. After 30 seconds, slowly exhale
4. Resume normal respiration

It is important to maintain an open glottis because this ensures continuous inspiratory effort during the performance of the technique. A closed glottis creates conditions for the Valsalva maneuver or simple breath-holding if the technique is performed incorrectly. If patients were observed performing the technique incorrectly, they were coached on correct performance. Following additional coaching, each patient was able to successfully complete the technique.

## Results

Following education and successful performance of the HAPI technique, each of the 21 patients in this sample reported immediate hiccup relief. No harms were noted, and no patients reported discomfort with the technique. Despite encountering initial challenges with performance of the technique, further coaching allowed each patient to eventually perform the technique correctly. There were no cases-including unreported cases-where the technique was performed but failed to cure hiccups.

A follow-up visit was conducted for the patient presenting with persistent hiccups. He was evaluated one week later and continued to be seen for routine contact over the following two years. During that time, he has reported no recurrence of persistent hiccups. He also reports that he has had several episodes of routine self-limited hiccups since initially using that HAPI technique, and he continues to use the technique with success.

Following their initial success, several individuals have reported successful re-use of the HAPI technique during subsequent episodes. A few have also reported teaching it to others and stated that it was successful in treating their hiccups as well. While these anecdotal reports are encouraging, information regarding recurrence was not systematically obtained. Follow-up for re-evaluation of hiccups was planned only with the patient who had intractable hiccups. Consequently, reliable data are not available on the success of the HAPI technique when used for recurrent episodes of hiccups.

## Discussion

The HAPI technique is a safe and simple intervention that is accessible to the majority of adult hiccup sufferers. It addresses two major targets for hiccup resolution, namely inhibition of the neurologic reflex arc and hypercapnia. It is safe and simple to perform, and was successful in all of the presented cases.

While the HAPI technique appears successful when used to treat self-limited hiccups, only a single patient with intractable hiccups was identified in this case series. The technique was successful in this patient, suggesting that this conservative treatment may be useful as a first-line option in this setting. Certainly the harms of attempting it are minimal compared with other methods such as medication, nerve blocks, and nerve stimulators [9,16].

Many patients faced the challenge of inadvertently closing the glottis during the prolonged inspiration step when learning the technique. Additionally, several subjects performed Valsalva during their initial attempts. Repeat education on correct performance of the HAPI technique was successful in addressing incorrect performance.

The technique was attempted on patients who were on average fairly healthy and young (the oldest patient was 64). It also was only used on individuals with spontaneous hiccups. It may not be as successful when used on patients with hiccups induced by surgery, tumors, or neurologic deficits [16-18]. Additionally, those with underlying lung conditions may find this technique difficult to perform given the constraints on limiting respiration for 30 seconds.

Every patient in this series was either an adult or older adolescent. It is unknown whether the HAPI technique would be as successful in older adults or younger children. At a minimum, it would not be expected to work with patients who are too young to follow the instructions of the technique. Likewise, patients with neurologic or cognitive deficits may not be capable of prolonged inspiratory effort.

Each patient was instructed to perform the technique for 30 seconds, though other durations could conceivably work with equal effectiveness. The impact of shorter or longer time periods on performance characteristics was not evaluated in this case series. Attempting the technique for longer or shorter time periods could be a subject for future study.

The nature of this study as a non-randomized retrospective case series likely introduces substantial bias, limiting the strength of the conclusions that can be drawn from it. For this reason, caution is advised in generalizing these findings. Further investigation with a more robust study design could help clarify the effectiveness or risks of the HAPI technique.

Given their sporadic, benign, and transient nature, it may be infeasible to perform any prospective investigation into the treatment of self-limited hiccups. There may be opportunity for cohort studies in patients with chronic or recurrent cases. The HAPI technique has the potential to spare medication and improve quality of life even if it is only successful at providing temporary relief.

## Conclusions

Hiccups are a common and often self-limited reflex phenomenon that can be distressing and disruptive to daily life. Prolonged hiccups can be caused by underlying medical conditions and may require medical treatment. While there are many home remedies for hiccup relief, these are not consistently effective and are not supported by strong clinical evidence. The HAPI technique is a simple and effective method that can be used for relief of hiccups that may complement other treatment options. Further research is needed to determine the underlying effectiveness of this technique in larger populations, in hopes to establish and disseminate a more tried and true method for hiccup relief.
